# Congenital Double Pylorus

**DOI:** 10.1155/2012/537697

**Published:** 2012-08-09

**Authors:** Ruvashni Naidoo, Bhugwan Singh

**Affiliations:** Department of General Surgery, School of Clinical Medicine, Nelson Mandela School of Medicine, University of Kwazulu-Natal, Private Bag X3, Congella, 4013 Durban, South Africa

## Abstract

The double pylorus is an uncommon finding and maybe congenital due to gastrointestinal duplication abnormality or more commonly secondary to peptic ulcer disease. The case we present is an elderly patient with mild dyspeptic symptoms who had an upper endoscopy as part of her investigative workup. The congenital double pylorus, being asymptomatic, may often go undetected. It is sometimes found incidentally on upper endoscopy, but needs no directed therapy. It is not associated with any specific complication.

## 1. Introduction

The double pylorus is a rare condition often found incidentally on endoscopy. It may be a complication of peptic ulcer disease or represent a developmental anomaly in an otherwise asymptomatic patient.

## 2. Case Presentation

**Figure 1:**
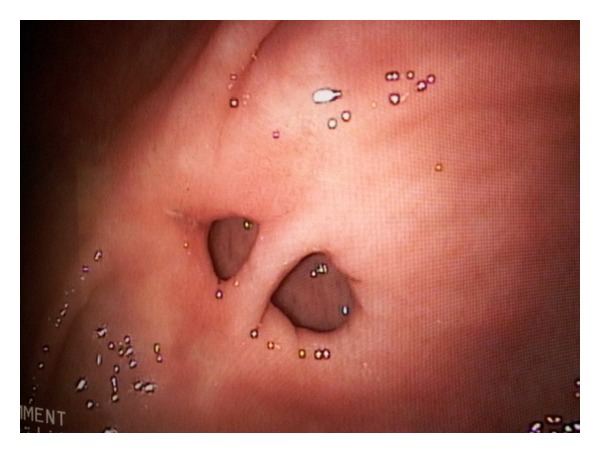
Endoscopic view demonstrating a double pylorus.

We present a 66-year-old female who presented to surgical outpatient department with mild epigastric pain. She denied any history of peptic ulcer disease or use of ulcerogenic drugs, such as NSAIDs. Clinical examination revealed an otherwise fit patient; the abdominal examination was normal. The endoscopy showed no evidence of peptic ulceration but demonstrated a double pylorus (Figure 1). The mucosa of the stomach as well as the duodenum appeared normal. The duodenum was easily entered via both pyloric channels. The patient was treated symptomatically and is now well. A follow-up endoscopy 6 weeks later demonstrated the same findings as the initial endoscopy.

## 3. Discussion

Double pylorus may be considered as a gastroduodenal fistula consisting of a short accessory channel between the distal stomach and the duodenal bulb, such that the gastric antrum and the duodenal bulb are connected by 2 openings separated by a septum or bridge of tissue [1]. This uncommon condition has a reported endoscopic incidence between 0.02% and 0.08% [1, 2]; the actual incidence of this abnormality is estimated to be between 0.06% and 0.4% [1, 3].

The double pylorus may occur as an exceedingly rare congenital anomaly or, relatively more commonly, secondary to peptic ulcer disease [1, 4]. The first congenital double pylorus was reported in 1971; since then congenital double pylorus has been rarely reported [3]. In the absence of evidence of peptic ulceration on endoscopy, as well as the negative history of peptic ulcer disease and dyspeptic symptoms or the use of NSAIDS, in the patient we report, a diagnosis of a congenital double pylorus was made.

The aetiology of congenital double pylorus has been attributed to a tubular duplication of the pylorus [5]. Whilst there are several theories proposed for gastrointestinal tract duplications, no one theory is adequate to explain these abnormalities throughout the gastrointestinal system. In an extensive literature review of all gastrointestinal tract duplications published in English literature, pyloric duplication was found in only 1 out of 281 cases reported [6]. This is a testament to the rarity of a congenital double pylorus.

It is widely accepted that the congenital double pylorus is largely asymptomatic and requires no intervention in most cases.
